# Anesthesiologists' Responses to an Email Request for Advice from an Unknown Patient

**DOI:** 10.2196/jmir.2.3.e16

**Published:** 2000-09-18

**Authors:** John Oyston

**Affiliations:** ^1^Department of AnesthesiaScarborough General HospitalCanada

**Keywords:** Internet, Email, Electronic Mail, Referral and Consultation, Medical History Taking, Quality of Health Care, Physician's Practice Patterns, Remote Consultation, Physician-Patient Relations, Professional-Patient Relations, Medical History Taking

## Abstract

**Background:**

People are using the Internet as a method of getting medical advice. Some Web sites include the email addresses of physicians, and some people are contacting these physicians for advice. As many patients undergo surgery on a "day surgery" basis, they often have no opportunity to ask anesthesiologists for advice before surgery; these patients may be more likely than other groups to use Internet email to ask questions. It seemed that it would be useful to find out what, if any, advice anesthesiologists would give in response to email from an unknown patient.

**Objective:**

To determine how anesthesiologists would respond to an email requesting advice about an anesthetic problem from an unknown patient.

**Methods:**

In February 1998, an email message was sent from a fictitious patient, using an email address created for this study, to 115 anesthesiologists whose email addresses were found on publicly accessible web sites. The message described the patient's problem with a previously administered anesthetic and requested advice about anesthesia for upcoming surgery. Responses were entered in a database and analyzed to determine the percentage of anesthesiologists who responded, and how helpful, accurate, and complete their advice was.

**Results:**

Fifty-eight responses were obtained from 108 valid email addresses (54% response rate). Of these, 78% were received within 48 hours. Eighty-three percent (83%) of respondents suggested contacting a local physician, 62% mentioned reviewing the old chart, and 41% suggested a specific diagnosis. None of the initial replies contained inaccurate advice, but only five responses were considered to be comprehensive. Ten percent (10%) included a disclaimer with the response. Eighty-three percent (83%) of replies were subjectively assessed as being friendly in tone.

**Conclusions:**

At present, patients who email an unknown anesthesiologist can expect to get a reply from over half. The advice is likely to be prompt, friendly, and to provide accurate and appropriate - but probably incomplete - advice.

## Introduction

*Note: An accompanying editorial, "A Question of Duty: Legal Issues Resulting from Physician Response to Unsolicited Patient Email Inquiries,"by P. Kuszler, MD, JD, has been published in this issue.*

Many patients are using the Internet as a source of medical information. Mainstream publications, such as "Consumers Reports" are teaching the public how to use the Web, Internet mailing lists, and email to get medical information and advice from the Internet [1].

New methods of communication between doctors and the public give rise to new practical, ethical, moral, and legal issues [2]. There are established guidelines for the use of email in established doctor-patient relationships [3]. Comparable guidelines in dealing with email from unknown members of the public are currently being proposed [4]. This issue was considered "a significant unresolved problem" by 62% of respondents to one survey [5].

One study published in the lay press [1] investigated eight web sites that invited medical questions. Only three sites (38%) responded, and two gave vague or unhelpful replies. Another study found that only ten of seventeen "cyberdocs" responded to a request for advice on a dermatological emergency, and that two gave questionable advice, suggesting that vitamins and herbal remedies would suffice [6]. These studies involved physicians who were setting themselves up to answer patient's questions, and yet they provided a very poor service.

This study involved sending email to physicians who have published an email address on a web site, but have not specifically requested email from patients. In a similar study, it was found that 18 out of 56 dermatologists were willing to offer a diagnosis for an unknown patient via email [5]. There has not been an equivalent study of anesthesiologists, or of any other medical specialty. As one Canadian anesthesiologist reports that he receives now about 50 email messages a week from unknown patients as the result of having his email address posted on a web site [7], it seemed appropriate to investigate how anesthesiologists respond to requests for medical advice from unknown patients, to determine if physicians in a different specialty were also willing and able to respond to patients' questions.

## Methods

To determine the response a patient would get from sending an email request for advice to an anesthesiologist, a fictitious patient email address was set up using HotMail. An email message asking for advice about an anesthetic problem was created, based on an actual email the author had received from a patient. In this message [see Box 1] the patient gave a history of requiring ventilation after a previous minor operation, and asked for advice about a future anesthetic. The history suggested that the patient had pseudocholinesterase deficiency, an inherited condition in which patients remain paralyzed for a prolonged period after the use of succinylcholine. This inheritable condition can be diagnosed by a simple blood test and treated by avoiding certain muscle relaxants.

The message was sent in February 1998 to 115 anesthesiologists whose email addresses were published on publicly accessible web sites. The addresses were found by searching English-language web pages listed at major anesthesia sites such as GASNet, the Anesthesiology section of The Mining Company(now About.com), and Anesthesia and Critical Care Resources on the Internet. Pages were searched for the "@" sign. Where the context made it clear that this was an anesthesiologist's email address, the address was used. Responses were read and analyzed for the presence or absence of certain types of statements, such as a disclaimer of responsibility, a diagnosis, specific points of advice, or suggested course of action. Data was entered into an EpiInfo 6.0 database [8] and analyzed using simple descriptive statistics.

A follow up message was sent to each responding anesthesiologist eight to ten days later, stating that the patient had been diagnosed with pseudocholinesterase deficiency and had had a successful anesthetic avoiding the use of succinylcholine [see Box 2].

**Fictitious patient request for help sent to 115 anesthesiologists**

Dear Doctor:

I would appreciate your advice about an anesthesia problem.

When I had my appendix out, I had to be put on a breathing machine after surgery. I was told this was because of a problem with the way my body handles anesthetic drugs.

Now I need surgery on my gall bladder and I am worried about the anesthetic. Can you give me any advice?

Thank you,

John Wilkinson

**Follow up message**

Dear Doctor:

Thanks for your reply to my request for anesthetic advice.

I thought you would like to know that a blood test showed I lacked an enzyme called "pseudocholinesterase" which makes me sensitive to "succinylcholine".

My anesthetist used a different drug and everything went fine.

Thanks again,

John Wilkinson

## Results

The results are summarized in Table 1. There were 65 replies to the 115 email requests for advice. Seven were messages saying the email address was invalid. These were excluded from further analysis, leaving 58 responses from 108 valid addresses, for a 54% response rate. Seventy-eight percent (78%) of replies were received within 48 hours, and all replies were received within five days.

Six respondents declined to give an opinion without more information. Ten asked "Who are you?", and six asked "Where are you?". Ten asked specific clinical questions, and seven invited further correspondence, including one who gave a fax number to which the patient's physician could forward the old medical record.

Forty-eight respondents (83% of replies) suggested the need to consult a physician. Forty-seven suggested an anesthetic consult, and five suggested contacting the family doctor. Fifteen responses stressed the need to arrange an early preoperative consultation. Thirty-six responses (62% of replies) mentioned the need to review the old chart.

Twenty-four responses (41% of replies) suggested a specific diagnosis. Of these, 88% mentioned succinylcholine, 67% specifically suggested avoiding succinylcholine, 58% suggested a blood test, 50% suggested other possible diagnoses, and 33% suggested that this could be a genetic problem.

Ten percent of respondents prefaced their remarks with a disclaimer, and one said that it was inappropriate to seek medical advice by email. None referred the patient to another resource, such as a web page or a journal article. Twenty-six (45%) of respondents offered reassurance that the problem could be dealt with safely. None of the initial replies contained inaccurate or inappropriate medical advice.

Only five of the responses were comprehensive, including the probable diagnosis, a mention of familial involvement, the possibility of a blood test, the need for an anesthetic consult, and the recommendation to avoid succinylcholine. The overall tone of the messages was subjectively assessed as being unfriendly in 3%, neutral in 14%, friendly in 78% and very friendly in 5% of replies.

A second email was sent to the 58 respondents, reporting the successful outcome of the surgery. This generated 26 responses (45%). Responses included: "Glad to hear all went well" (15 responses); "Document problem/tell others" (9); "Consider a MedicAlert bracelet" (7); "Thanks for letting me know" (5); "Beware of Mivacurium as well as succinylcholine" (4); "Have family members tested" (4); "It is not a problem now that it is diagnosed" (4); and "Avoid Atracurium" (1). This last was the only false piece of advice in any response to the survey.

**Table 1:** Results Summary and comparison with an earlier study

	**Current Study**	**Eysenbach et al [5]**
**Question**	Requesting advice about an upcoming anesthetic after a previous problem	Requesting advice about a dermatological emergency
**Study Group**	115 anesthesiologists (108 valid email addresses) 58 responses	58 dermatologists (56 valid email addresses) 29 responses
**Advised To See MD**	48/58 (83%)	27/29 (93%)
**Specific Diagnosis**	24/58 (41%) All "correct"	18/29 (62%) One "incorrect"
**Specific Treatment**	18/24 (67%)	5/18 (28%)
**Refused To Give Advice**	6/58 (10%)	2/29 (7%)

## Discussion

As the public becomes more aware of medical resources on the Internet, some patients are emailing unknown physicians to request medical advice. This produces an ethical and legal dilemma, as the recipient of the message has to balance a natural and desirable human response to offer help to someone who has a problem against the medical, legal, and ethical pitfalls of providing advice to an unknown person, without access to the past medical history, a physical examination, or laboratory data [4]. Usually the patient expects advice for free, but even giving free advice exposes the physician to the possibility of legal action for malpractice or for practicing in a jurisdiction in which he or she has no license. There is no consensus as to how best to deal with this issue.

A recent survey of dermatology web sites revealed that 24% attempted to answer patients' questions individually, 27% did not usually respond and 24% usually sent a form letter [6]. As part of the same study, the authors also sent a fictitious email request from an immunocompromised "patient" who required urgent treatment of herpes zoster to 58 dermatologists whose email address appeared in dermatology web sites. Their study had results similar to this one [see Table 1]. Their response rate was 52% (compared to 54% in this study), "usually within 1 - 2 days" (compared to 78% in 48 hours). Ninety-three percent (93%) of their responses said, "See a local physician" (compared to 83% in this study). Sixty-two percent (62%) of dermatologists suggested a diagnosis, compared with 41% of anesthesiologists. As the scenarios are not comparable, it is not possible to determine if anesthesiologists and dermatologists differ in their propensity to provide medical advice based on information in an unsolicited email.

In the current study, just over half (54%) of anesthesiologists responded to the email request, and of these 41% suggested a specific diagnosis. Only a minority were concerned enough to add a disclaimer to their comments. They provided advice which was timely and appropriate, in a friendly manner, but which was rarely complete. From a patient's perspective, emailing an unknown anesthesiologist for advice about an anesthetic problem appears to be a useful way of acquiring information, even if the majority of responses included the suggestion of consulting a local anesthesiologist and having him/her review the old chart.

It is estimated that 28 million people undergo surgery each year in the USA alone [9]. Many of these patients will not meet an anesthesiologist until the day of surgery, so it would not be surprising if more of them start to look for answers to their anesthesiology questions on the Internet.

Ideally, patients seeking advice about anesthesia should be able to contact a local anesthesiologist in person, so that the situation can be reviewed in conjunction with medical records and laboratory data. Email may be appropriate for follow up, for example for the patient to provide extra details, or for the anesthesiologist to forward laboratory results. However, some patients are likely to want second opinions, or to ask advice of someone assumed to be an expert in a specific field (such as the author of a relevant web page). This survey suggests that if they do so, they stand a reasonable chance of getting a prompt, friendly reply, which is likely to be valid, but may be incomplete.
